# Concomitant Alpha- and Gamma-Sarcoglycan Deficiencies in a Turkish Boy with a Novel Deletion in the Alpha-Sarcoglycan Gene

**DOI:** 10.1155/2014/248561

**Published:** 2014-06-22

**Authors:** Gulden Diniz, Hulya Tosun Yildirim, Sarenur Gokben, Gul Serdaroglu, Filiz Hazan, Kanay Yararbas, Ajlan Tukun

**Affiliations:** ^1^Neuromuscular Diseases Centre, Tepecik Research Hospital, Kibris Sehitleri Caddesi 51/11, Alsancak, 35220 Izmir, Turkey; ^2^Pathology Department, Dr. Behcet Uz Children's Research Hospital, 35210 İzmir, Turkey; ^3^Pediatric Neurology Department, Faculty of Medicine, Ege University, 35100 İzmir, Turkey; ^4^Medical Genetics Department, Dr. Behcet Uz Children's Research Hospital, 35210 Izmir, Turkey; ^5^Medical Genetics Department, Duzen Laboratories, Istanbul, Turkey; ^6^Medical Genetics Department, Duzen Laboratories, Ankara, Turkey; ^7^Medical Genetics Department, Faculty of Medicine, Ankara University, 06100 Ankara, Turkey

## Abstract

Limb-girdle muscular dystrophy type 2D (LGMD-2D) is caused by autosomal recessive defects in the alpha-sarcoglycan gene located on chromosome 17q21. In this study, we present a child with alpha-sarcoglycanopathy and describe a novel deletion in the alpha-sarcoglycan gene. A 5-year-old boy had a very high serum creatinine phosphokinase level, which was determined incidentally, and a negative molecular test for the dystrophin gene. Muscle biopsy showed dystrophic features. Immunohistochemistry showed that there was diminished expression of alpha- and gamma-sarcoglycans. DNA analysis revealed a novel 7 bp homozygous deletion in exon 3 of the alpha-sarcoglycan gene. His parents were consanguineous heterozygous carriers of the same deletion. We believe this is the first confirmed case of primary alpha-sarcoglycanopathy with a novel deletion in Turkey. In addition, this study demonstrated that both muscle biopsy and DNA analysis remain important methods for the differential diagnosis of muscular dystrophies because dystrophinopathies and sarcoglycanopathies are so similar.

## 1. Introduction

Limb girdle muscular dystrophy type 2D (LGMD-2D) is an autosomal recessive muscular disease caused by genetic defects in sarcolemmal alpha sarcoglycan (*α*-SGC) glycoprotein. Alpha-SGC or adhalin, one of the four sarcoglycans (SGCs), is essential for membrane integrity during muscle contraction and provides a scaffold for important signaling molecules [1–3]. Alpha-SGC is encoded by the sarcoglycan alpha gene (SGCA) located on chromosome 17q21 [1, 4]. LGMD-2D predominantly affects proximal muscles around the scapular and the pelvic girdles. LGMD-2D has a very heterogeneous phenotype. The age of onset, rate of progression, and the severity of disease can vary between and also within affected families. The most clinically severe course is generally observed when the sarcolemmal *α*-SGC is totally absent whereas milder phenotypes are observed when residual proteins are present [1–4]. Interestingly, a mutation in any SGC gene can lead to a reduction or absence of the other SGCs [4–7]. It was previously reported that the SGCA gene must be evaluated first if there is a concomitant absence of *α*-SGC and gamma- (*γ*-) SGC [4].

The differential diagnosis for LGMD-2D includes Duchenne and Becker muscular dystrophies (DMD/BMD) and it is impossible to reach a diagnosis on clinical grounds alone. Therefore, immunohistochemical staining of a muscle biopsy and molecular genetic analysis are mandatory for the correct diagnosis [3, 5, 8, 9]. In this report, the patient's genotype was a previously unknown 7 bp deletion in exon 3. This finding adds to the growing spectrum of mutations in the alpha-sarcoglycan gene. Finally, we also discuss important considerations in the differential diagnosis of the muscular dystrophies.

## 2. Case Report

A 5-year-old boy had second degree consanguineous parents from Turkey without an ancestral history of neuromuscular disorders. There were no complications during pregnancy, and antenatal signs of muscular disorders such as polyhydramnios and reduced fetal movements were not noted. Cognitive and motor development was normal. At the time of presentation, his previously undetected mild muscle weakness was predominantly proximal. Deep tendon reflexes were present and he had no contractures. He was walking normally but he had mild difficulty when climbing stairs and running. Pulmonary function tests were normal. His creatinine phosphokinase (CPK) levels were between 9000 and 15000 units per liter (normal < 250 U/L), and there were myopathic changes on electromyography. Because of the very high CPK level, muscular dystrophy was suspected and, after informed consent, samples were obtained for histopathology, immunohistochemistry, and molecular genetics testing.

A muscle biopsy specimen from the left gastrocnemius muscle of the patient was frozen in isopentane that was precooled to −160°C in liquid nitrogen. Cryosections were immunostained for dystrophin using a polyclonal antibody (Neomarkers), with a monoclonal spectrin antibody (Novocastra) as a control. A neonatal myosin heavy chain (Neonatal myosin, Novocastra) antibody was used for the identification of pathological immature myofibers. SGCs were detected with anti-*α*-, -*β*-, -*δ*-, and -*γ*-SGC antibodies (Novocastra).

Peripheral blood specimens were collected from the proband and parents. Genomic DNA was extracted from whole blood using a commercial DNA extraction kit (QiaGen, USA) following the standard manufacturer's protocol. The concentration of sample DNA was determined by a NanoDrop spectrophotometer (NanoDrop Technologies, Wilmington, DE). The exon regions and flanking short intronic sequences of the SGCA gene were amplified using polymerase chain reaction (PCR), followed by direct sequencing of the PCR products (ABI, US) (NCBI Reference Sequence: NG_008889.1). Hitherto reported genetic abnormalities in LGMD-2D are listed in Table 1.

## 3. Results

**Figure 1:**
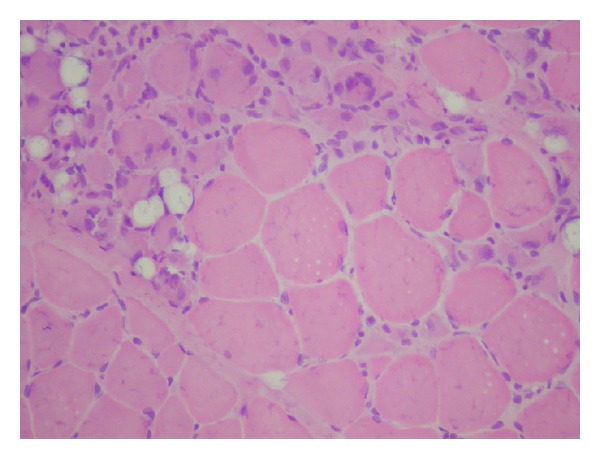
Differences in the size and shape of myofibers as well as regeneration (HEx 200).

**Figure 2:**
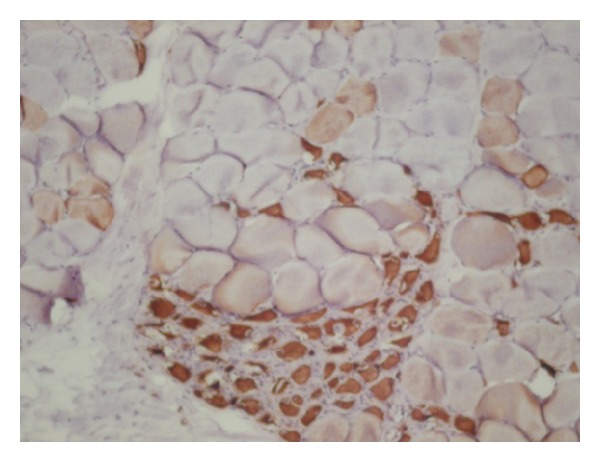
Immature pathological fibers visualized with anti-neonatal myosin antibody staining (DABx 100).

**Figure 3:**
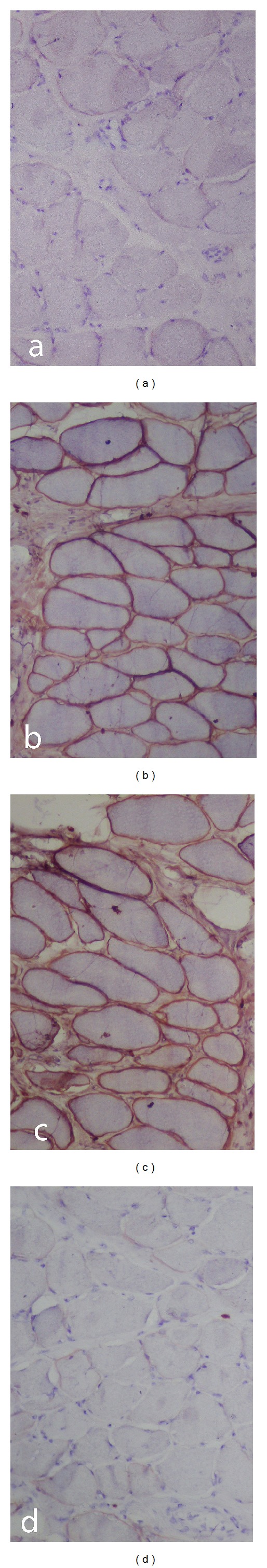
Diffuse absence of sarcolemmal *α*-SGC (a) and *γ*-SGC (d) expression and normal *β*-SGC (b) and *δ*-SGC (c) expression (DABx 200).

The muscle biopsy showed dystrophic changes like contraction, regeneration (Figure 1), degeneration, necrosis, nuclear internalization, and fibrosis. In addition, many pathological immature myofibers were visualized using the neonatal myosin staining (Figure 2). Based on immunostaining, dystrophin and spectrin expressions were normal. Except for isolated deficient fibers, beta (*β*) sarcoglycan and delta (*δ*) sarcoglycan were present at normal levels, whereas *α*-SGC and *γ*-SGC were diffusely absent (Figure 3).

**Figure 4:**
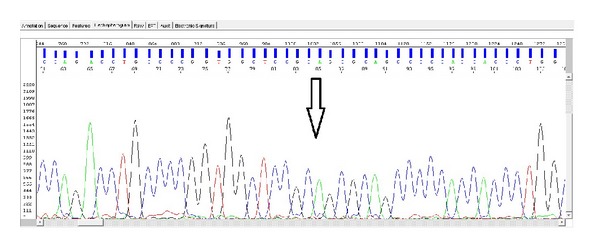
Proband exon 3 homozygous del TACACCC site.

**Figure 5:**
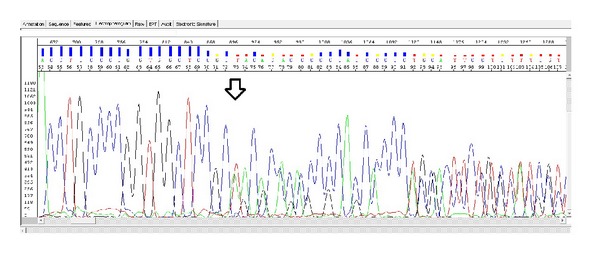
Maternal heterozygous del TACACCC site.

**Figure 6:**
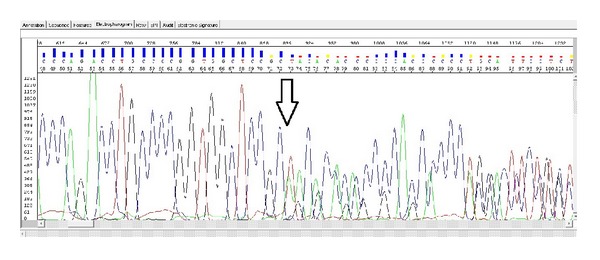
Paternal heterozygous del TACACCC site.

Based on analysis of the proband, we have identified a previously undetermined homozygous 7 bp deletion in exon 3 (Figure 4). A similar heterozygous deletion was found in both parents (Figures 5 and 6). Location of this deletion was also indicated in Table 1. In addition, there were no abnormalities in the dystrophin gene and the other sarcoglycan genes (SGCB, SGCD, and SGCG) in the patient and his parents.

## 4. Discussion

Human SGCA cDNA from a human skeletal muscle library was isolated and sequenced in 1993. This gene consisted of 10 exons. The protein product of SGCA gene consisted of 387 amino acids with an extracellular N-terminus, a transmembrane domain, and an intracellular C-terminus. Northern blot analysis showed that human adhalin mRNA was expressed at the highest levels in skeletal muscle. It was also expressed in cardiac muscle and in the lung, but at much lower levels. On the contrary, adhalin mRNA was not detected in the brain. It was also reported that the adhalin mRNA from cardiac muscle was shorter relative to skeletal muscle and that the base sequence encoding the transmembrane domain was absent. It is known that LGMD-2D primarily affects skeletal muscles while brain and peripheral nerve functions are largely preserved. Briefly, the less severe cardiac dysfunction and lack of mental retardation in patients with LGMD-2D may be explained by the lower expression of *α*-SGC in cardiac muscle and the absence of adhalin expression in the brain [1, 3, 10]. In the patient described in this report, we did not find clinical evidence of cardiac involvement, decreased intellectual capacity, or denervation (as demonstrated by electromyography). The course of the disease in this case suggests that this novel deletion may cause a milder phenotype of LGMD-2D despite the diffuse absence of *α*-SGC and *γ*-SGC.

Immunohistochemical analysis of sarcolemmal proteins in muscle biopsies like dystrophin, SGCs, merosin, and dysferlin is an important part of the diagnostic evaluation of patients with muscular dystrophy. Reduced or absent sarcolemmal expression of one of the 4 SGCs can be found in patients with any type of LGMDs and also in patients with dystrophinopathies. It has previously been suggested that different patterns of SGC expression could predict the primary genetic defect and that genetic analysis could be directed by these patterns [5–8]. However, Klinge et al. [9] reported that residual SGC expression could be highly variable and an accurate prediction of the genotype could not be achieved. Babameto-Laku et al. [4] also determined that the concomitant absence of *α*-SGC and *γ*-SGC expression was caused by defects in the SGCA gene. Therefore, they recommended using antibodies against all four SGCs for immunoanalysis of skeletal muscle sections. Similarly, a concomitant reduction in dystrophin and any of the SGCs may illustrate the importance of considering coexisting dystrophinopathies in patients with sarcoglycan-deficient LGMD [9–13]. For this reason, it is not easy to decide whether the disease is a dystrophinopathy with defective expressions of SGCs or a LGMD with defective expression of dystrophin. However, in the patient described in this report, dystrophinopathies, such as DMD and BMD, were ruled out because the expression of sarcolemmal dystrophin was diffusely present and molecular tests for dystrophin gene were normal.

At present, more than 70 mutations have been reported in the SGCA gene that cause changes in the *α*-SGC glycoprotein. Approximately a two-thirds of mutations are missense mutations that generate a complete protein with a single residue substitution, whereas other mutations like nucleotide replacements, duplications, deletions, or insertions produce truncated, incomplete, or anomalous proteins. Almost all missense mutations map to the extracellular domain which is a critical region for the organization of SGCs and their association with dystroglycan. Only a single missense mutation maps to the intracellular domain and causes LGMD-2D in homozygous cases. Similarly, two mutations caused by deletions generate a normal extracellular portion of *α*-SGC and truncated intracellular tails. At present, there is no data about the intracellular tail of the *α*-SGC protein and its function [1, 10–14]. In the family described in this report, we discovered a novel deletion in the TACACCC site of exon 3 that would cause a frame-shift mutation. The past literature highlights that the prediction of pathological consequences associated with different mutations of SGCA gene is very complex. It is not clear whether this novel deletion generates a severe disease phenotype or whether it also has additional, undetermined consequences.

Patients with any of the LGMDs may be clinically indistinguishable from those with the primary dystrophinopathies. It is likely that the prevalence of LGMD is underestimated and a number of male patients are incorrectly diagnosed with DMD or BMD [13]. A definitive diagnosis rests on performing the appropriate immunohistochemical examination as well as doing a molecular analysis. A normal dystrophin staining pattern should be seen as well as an autosomal recessive mode of inheritance. In contrast, the patients with dystrophinopathies may show variable findings from a normal to a regional absence or a mosaic pattern of sarcolemmal staining with anti-SGCs antibodies which correspond to an abnormal organization of the cell-membrane-associated dystrophin glycoprotein complex. Therefore, it is necessary to perform a careful examination of the immunohistochemical staining as well as a genetic study in order to make the correct diagnosis.

In summary, this report describes a novel deletion that adds to the growing list of defects associated with LGMD-2D and further emphasizes the importance of systematic analysis of all related genes, instead of limiting the analysis to the one SGC gene that is hypothesized to be the cause of the abnormalities. In this study, we also highlight the complexity of staining patterns associated with sarcolemmal proteins and the importance of careful analysis of this staining pattern in order to narrow the differential diagnosis of muscular dystrophies.

## Figures and Tables

**Table 1:** Nucleotide and amino acid sequences of *α*-SGC gene.

1 1	ATG Met	GCT Ala	GAG Glu	ACA Thr	CTC Leu	TTC Phe	TGG Trp	ACT Thr	CCT Pro	CTC Leu	CTC Leu	GTG Val	GTT Val	CTC Leu	CTG Leu

46 16	GCA Ala	GGG Gly	CTG Leu	GGG Gly	GAC Asp	ACC Thr	GAG Glu	GCC Ala	CAG Gln	CAG Gln	ACC Thr	ACG Thr	CTA Leu	CAC His	CCA Pro^#^

91 31	CTT Leu^#^	GTG Val	GGC Gly	CGT Arg^#^	GTC Val	TTT Phe	GTG Val	CAC His	ACC Thr	TTG Leu	GAC Asp	CAT His	GAG Glu	ACG Thr	TTT Phe

136 46	CTG Leu	AGC Ser	CTT Leu	CCT Pro	GAG Glu	CAT His	GTC Val	GCT Ala^#^	GTC Val	CCA Pro	CCC Pro	GCT Ala	GTC Val	CAC His	ATC Ile

181 61	ACC Thr	TAC Tyr^#^	CAC His	GCC Ala	CAC His	CTC Leu	CAG Gln	GGA Gly^#^	CAC His	CCA Pro	GAC Asp	CTG Leu	CCC Pro	CGG Arg^#^	TGG Trp

226 76	CTC Leu	CGC Arg^#^	**TAC** Tyr^##^	**ACC** Thr^##^	**C**AG Gln^##^	CGC Arg^#^	AGC Ser	CCC Pro	CAC His	CAC His	CCT Pro	GGC Gly	TTC Phe	CTC Leu^#^	TAC Tyr^#^

271 91	GGC Gly^#^	TCT Ser	GCC Ala^#^	ACC Thr	CCA Pro	GAA Glu	GAT Asp^#^	CGT Arg^#^	GGG Gly	CTC Leu	CAG Gln	GTC Val	ATT Ile^#^	GAG Glu	GTC Val

316 106	ACA Thr	GCC Ala	TAC Tyr	AAT Asn	CGG Arg^#^	GAC Asp	AGC Ser	TTT Phe	GAT Asp	ACC Thr	ACT Thr	CGG Arg	CAG Gln	AGG Arg	CTG Leu

361 121	GTG Val	CTG Leu	GAG Glu	ATT Ile^#^	GGG Gly	GAC Asp	CCA Pro	GAA Glu	GGC Gly	CCC Pro	CTG Leu	CTG Leu	CCA Pro	TAC Tyr	CAA Gln

406 136	GCC Ala	GAG Glu^#^	TTC Phe	CTG Leu^#^	GTG Val	CGC Arg^#^	AGC Ser	CAC His	GAT Asp	GCG Ala	GAG Glu	GAG Glu	GTG Val	CTG Leu	CCC Pro

451 151	TCA Ser	ACA Thr	CCT Pro	GCC Ala	AGC Ser	CGC Arg	TTC Phe	CTC Leu^#^	TCA Ser	GCC Ala	TTG Leu	GGG Gly	GGA Gly	CTC Leu	TGG Trp

496 166	GAG Glu	CCC Pro	GGA Gly	GAG Glu	CTT Leu	CAG Gln	CTG Leu	CTC Leu^#^	AAC Asn	GTC Val^#^	ACC Thr	TCT Ser	GCC Ala	TTG Leu	GAC Asp

541 181	CGT Arg^#^	GGG Gly	GGC Gly	CGT Arg	GTC Val	CCC Pro	CTT Leu	CCC Pro	ATT Ile	GAG Glu	GGC Gly	CGA Arg	AAA Lys	GAA Glu	GGG Gly^#^

586 196	GTA Val^#^	TAC Tyr	ATT Ile	AAG Lys	GTG Val	GGT Gly	TCT Ser	GCC Ala	TCA Ser	CCT Pro^#^	TTT Phe	TCT Ser	ACT Thr^#^	TGC Cys	CTG Leu

631 211	AAG Lys	ATG Met	GTG Val	GCA Ala	TCC Ser^#^	CCC Pro	GAT Asp	AGC Ser	CAC His	GCC Ala	CGC Arg^#^	TGT Cys	GCC Ala	CAG Gln^#^	GGC Gly

676 226	CAG Gln	CCT Pro^#^	CCA Pro^#^	CTT Leu	CTG Leu	TCT Ser	TGC Cys^#^	TAC Tyr	GAC Asp	ACC Thr	TTG Leu	GCA Ala	CCC Pro^#^	CAC His	TTC Phe

721 241	CGC Arg	GTT Val^#^	GAC Asp	TGG Trp	TGC Cys	AAT Asn	GTG Val^#^	ACC Thr	CTG Leu	GTG Val	GAT Asp	AAG Lys	TCA Ser	GTG Val	CCG Pro

766 256	GAG Glu	CCT Pro	GCA Ala	GAT Asp	GAG Glu	GTG Val	CCC Pro^#^	ACC Thr	CCA Pro	GGT Gly	GAT Asp	GGG Gly	ATC Ile	CTG Leu	GAG Glu

811 271	CAT His	GAC Asp^#^	CCG Pro	TTC Phe	TTC Phe	TGC Cys	CCA Pro	CCC Pro	ACT Thr	GAG Glu	GCC Ala	CCA Pro	GAC Asp	CGT Arg^#^	GAC Asp

856 286	TTC Phe	TTG Leu	GTG Val	GAT Asp	GCT Ala	CTG Leu	GTC Val	ACC Thr	CTC Leu	CTG Leu	GTG Val	CCC Pro	CTG Leu	CTG Leu	GTG Val

901 301	GCC Ala	CTG Leu	CTT Leu	CTC Leu	ACC Thr	TTG Leu	CTG Leu	CTG Leu	GCC Ala	TAT Tyr	GTC Val	ATG Met^#^	TGC Cys	TGC Cys	CGG Arg

946 316	CGG Arg	GAG Glu	GGA Gly	AGG Arg	CTG Leu	AAG Lys	AGA Arg	GAC Asp	CTG Leu	GCT Ala	ACC Thr	TCC Ser	GAC Asp	ATC Ile	CAG Gln

991 331	ATG Met	GTC Val	CAC His	CAC His	TGC Cys	ACC Thr	ATC Ile	CAC His	GGG Gly	AAC Asn	ACA Thr	GAG Glu	GAG Glu	CTG Leu	CGG Arg

1036 346	CAG Gln	ATG Met	GCG Ala	GCC Ala	AGC Ser	CGC Arg	GAG Glu	GTG Val	CCC Pro	CGG Arg	CCA Pro	CTC Leu	TCC Ser	ACC Thr	CTG Leu

1081 361	CCC Pro	ATG Met	TTC Phe	AAT Asn	GTG Val	CAC His	ACA Thr	GGT Gly	GAG Glu	CGG Arg	CTG Leu	CCT Pro	CCC Pro	CGC Arg	GTG Val

1126 376	GAC Asp	AGC Ser	GCC Ala	CAG Gln	GTG Val	CCC Pro	CTC Leu	ATT Ile	CTG Leu	GAC Asp	CAG Gln	CAC His	TGA Ter

*Note the previously determined missense mutations marked with #. The present deletion was marked with ## and bold letters.
